# Germline gene editing and the precautionary principle

**DOI:** 10.1111/bioe.12609

**Published:** 2019-06-27

**Authors:** Julian J. Koplin, Christopher Gyngell, Julian Savulescu

**Affiliations:** ^1^ Biomedical Ethics Research Group Murdoch Children's Research Institute Melbourne Victoria Australia; ^2^ Melbourne Law School University of Melbourne Melbourne Victoria Australia; ^3^ Department of Paediatrics University of Melbourne Melbourne Victoria Australia; ^4^ Faculty of Philosophy Oxford Uehiro Centre for Practical Ethics Oxford United Kingdom of Great Britain and Northern Ireland

**Keywords:** gene editing, germline modification, precautionary principle, reproductive ethics, sufficientarianism

## Abstract

The precautionary principle aims to influence decision‐making in contexts where some activity poses uncertain but potentially grave threats. This perfectly describes the controversy surrounding germline gene editing. This article considers whether the precautionary principle should influence how we weigh the risks and benefits of human germline interventions, focusing especially on the possible threats to the health of future generations. We distinguish between several existing forms of the precautionary principle, assess their plausibility and consider their implications for the ethics of germline modification. We also offer a novel form of the precautionary principle: the sufficientarian precautionary principle. Some plausible versions of the precautionary principle recommend placing somewhat greater weight on avoiding threats to future generations than on achieving short‐term benefits. However, no plausible versions of the precautionary principle entail that we should outright reject the use germline gene editing in human reproduction and some, such as the sufficientarian version, might endorse its use.

## 1 INTRODUCTION

The recent development of gene editing technologies such as CRISPR‐Cas9 has revolutionized genetic engineering. Gene editing techniques are more efficient, more precise and less expensive than older methods of genetic modification. This has opened up a range of new applications for genetic engineering, the most controversial of which is the potential to use germline gene editing (GGE) in human reproduction.1
Gene editing technologies could also be used in somatic cell therapy, synthetic biology, the genetic modification of plants and livestock, and pest eradication. These other areas of application raise bioethical issues that arguably deserve greater attention than they have received to date. Caplan, A. L., Parent, B., Shen, M. & Plunkett, C. (2015). No time to waste: The ethical challenges created by CRISPR. *EMBO reports, 16*(11), 1421–1426.10.15252/embr.201541337PMC464149426450575



Recent events have brought the ethics of GGE to the forefront of public attention. In November 2018, He Jiankui of Shenzhen University claimed to have used CRISPR to edit the genes of twin girls, Lulu and Nana.2
Yong, E. (2018). *A reckless and needless use of gene editing on human embryos. The Atlantic* 26 Nov. Retrieved from https://www.theatlantic.com/science/archive/2018/11/first-gene-edited-babies-have-allegedly-been-born-in-china/576661/

 If these claims are true, GGE has already been used in human reproduction. He's experiment has been roundly condemned, and rightly so; it is widely agreed that gene editing technologies are still too unsafe for human trials.3
National Academies of Sciences, Engineering, and Medicine. (2017). *Human genome editing: Science, ethics, and governance *(Chapter 5). Washington, DC: National Academies Press.28796468
 However, while the use of GGE in human reproduction is almost definitely premature, we still need to ask whether intervening in the human germline could ever be ethically permissible. The fact that experiments with heritable GGE have apparently already been conducted suggests we will need to answer this question sooner rather than later.

The potential benefits of GGE for reproduction are threefold. In the short term, GGE may allow couples to have a genetically related child without passing on genetic disease, including circumstances where it is not possible to select an unaffected embryo using a pre‐implantation genetic diagnosis. This includes cases where individuals are homozygotes for dominant conditions like Huntington's disease, or when dominant de novo disease‐causing mutations develop in sperm or egg cells. Secondly, relative to genetic selection, GGE could improve the health of future generations by reducing the frequency of recessive genetic mutations.4
Gyngell, C. (2017). Gene editing and the health of future generations. *Journal of the Royal Society of Medicine,* *110*(7), 276–279.10.1177/0141076817705616PMC552425728445654
 Even when a pre‐implantation genetic diagnosis can be used to avoid monogenic or chromosomal diseases in a couple's offspring, in some cases all available embryos may carry recessive mutations that do not cause disease but would increase the risk of disease to future generations. Alternatively, they could carry many normal alleles that are associated with higher than normal risks of common diseases (such as APOE4, which elevates the risk of Alzheimer's disease). By comparison, GGE could be used to remove *all* disease‐causing genes from the embryo, or genes associated with elevated risk, reducing the incidence of such diseases in the next and future generations.5
Gyngell, C., Douglas, T., & Savulescu, J. (2017). The ethics of germline gene editing. *Journal of Applied Philosophy, 34*(4), 498–513.10.1111/japp.12249PMC557399228919655
 It would not be possible to select against such large numbers of disease‐causing or disadvantageous genes without very large numbers of embryos.6
Bourne, H., Douglas T., & Savulescu, J. (2012). Procreative beneficence and in vitro gametogenesis. *Monash Bioethics Review, 30*(2), 29–48.10.1007/BF03351338PMC359089923409535
 Thirdly, GGE could potentially be used to endow children with ‘protective' genes that reduce the risk of common diseases such as heart disease, cancer and diabetes, potentially benefitting both the children themselves and their descendants.7
Lander, E. S. (2015). Brave new genome. *New England Journal of Medicine, 373*, 5–8.10.1056/NEJMp150644626039524



Three safety risks weigh against these potential benefits.8
Ibid.; Bosley, K. S., Botchan, M., Bredenoord, A. L., Carroll, D., Charo, R. A., Charpentier, E., … Zhou, Q. (2015). CRISPR germline engineering – the community speaks. *Nature Biotechnology, *33, 478–487. For the purposes of this article, we are bracketing concerns about eugenics, human dignity and human enhancement, as well as the practical difficulties of designing strategies for intergenerational monitoring.10.1038/nbt.322725965754
 Firstly, GGE may make unintended changes to the germline through off‐target mutations that may be missed in any safety checks that are performed. The harmful effects of these off‐target mutations may not be apparent until the recipient reaches old age, and could therefore potentially reverberate across future generations. Secondly, given the limitations of our knowledge of human genetics, there is a possibility that the intended genome edits themselves may cause unanticipated harms that, again, might reverberate across future generations. Thirdly, genetic variants used to decrease risk for some diseases may inadvertently increase risks for others, potentially rendering the recipients of these changes (and their descendants) vulnerable to future health threats.

These three risks are widely seen an important reason against pursuing GGE for the prevention of disease, and indeed are sometimes taken as a reason to reject GGE outright. In a widely cited commentary in *Nature*, Lanphier and colleagues argue that GGE is ‘dangerous and ethically unacceptable' because germline changes ‘could have unpredictable effects on future generations'.9
Lanphier, E., Lanphier, E., Urnov, F., Haecker, S. E., Werner, M. & Smolenski, J. (2015). Don't edit the human germ line. *Nature News, 519*(7544), 410.10.1038/519410a25810189
 Baltimore and colleagues, writing in *Science*, likewise discourage GGE because of the potential for harmful unintended consequences, especially those that might occur across generations.10
Baltimore, D., Berg, B., Botchan, M., Carroll, D., Charo, R. A., Church, G., … Yamamoto, K. R. (2015). A prudent path forward for genomic engineering and germline gene modification. *Science, 348*(6230), 36–38.10.1126/science.aab1028PMC439418325791083
 Similarly, in explaining the US National Institutes of Health's decision not fund GGE research, Francis Collins has noted that GGE poses ‘serious and unquantifiable safety issues'.11
Collins F. (2015). *Statement on NIH funding of research using gene‐editing technologies in human embryos* National Institutes of Health. Retrieved from https://www.nih.gov/about-nih/who-we-are/nih-director/statements/statement-nih-funding-research-using-gene-editing-technologies-human-embryos

 Although CRISPR‐Cas9 may be a new technology, these concerns over GGE are not. Over 15 years ago, Annas and colleagues argued for an international ban on human germline modification, partly on the ground that such modifications could endanger the human species.12
Annas, G. J., Andrews, L. B., & Isasi, R. M. (2002). Protecting the endangered human: toward an international treaty prohibiting cloning and inheritable alterations. *American Journal of Law & Medicine, 28*, 151–178. Interestingly, in addition to the risks outlined above, Annas and colleagues worry that mutual animosity between unedited humans and genetically enhanced ‘posthumans' may lead to the genocide of one group at the hands of the other. This concern has largely faded to the background of more recent discussions of GGE, and we do not explicitly discuss it in this article.12197461



What should we make of GGE's risks to future generations? The mere fact that there are risks does not show that we should reject GGE *tout court*, for it is sometimes worth taking a risky course of action if the potential benefits are great enough – and in the case of GGE, the potential benefits are significant. It might be thought that the risks entailed by GGE are incalculably greater than the potential benefits because these risks, if realized, affect an indefinite number of future generations. However, even bracketing general concerns regarding this style of argument,13
See, e.g. Munthe, C. (2019). The black hole challenge: Precaution, existential risks and the problem of knowledge gaps. *Ethics, Policy & Environment.* 10.1080/21550085.2019.1581415.
 the fact that the potential harms of GGE would affect an indefinitely large number of generations does not show that these risks necessarily outweigh the benefits. This is because some of the benefits of GGE – such as the promotion of health – would also affect an indefinite number of future generations. It might be thought that the risks to future generations are especially weighty because these future generations did not *consent* to having these risks imposed on them. However, future generations are also unable to consent to any of the myriad decisions we routinely make that affect the world future generations will be born into and the genes they will inherit.14
Gyngell et al., op. cit., note 5; Harris, J. (2015). Germline manipulation and our future worlds. *American Journal of Bioethics,15*(12), 30–34.10.1080/15265161.2015.110416326632358
 Finally – and perhaps most promisingly – it might be thought that because GGE impose serious and difficult‐to‐predict risks, it would be ruled out by (some plausible version of) the precautionary principle.

This article considers what role, if any, the precautionary principle should play in our ethical evaluation of GGE. We first distinguish negative precautionary principles (that reject certain kinds of arguments against precautionary policies) and positive precautionary principles (that prescribe rules for decision‐making in the face of potentially grave threats). We then trace the implications of various plausible versions of the precautionary principle for the use of GGE in human reproduction. Existing work on this topic tends to assume that the precautionary principle would either weigh GGE or rule it out altogether.15
See, e.g. Annas et al., op. cit., note 12; Peters, T. (2015). CRISPR, the precautionary principle, and bioethics. *Theology and Science, 13*(3), 267–270; Goldim, J. R. (2015). Genetics and ethics: A possible and necessary dialogue. *Journal of Community Genetics, 6*(3), 193–196; Smith, K. R., Chan, S., & Harris, J. (2012). Human germline genetic modification: Scientific and bioethical perspectives. *Archives of Medical Research,* *43*(7), 491–513. Not all these commentators welcome the conclusion that we should reject GGE. For example, Smith and colleagues reject the precautionary principle precisely because it would prevent us from realizing the potential gains of GGE.10.1016/j.arcmed.2012.09.00323072719
 We argue that this conclusion is too quick. There are unresolved questions about how the various plausible versions of the precautionary principle should be applied to GGE. There are also unresolved questions about whether we ought to take greater precaution against the risks entailed by GGE or the risks entailed by *failing* to pursue GGE. The precautionary principle, then, may have a legitimate role to play in our ethical evaluation of GGE. But exactly what position it would support remains to be seen.

## 2 VARIETIES OF PRECAUTIONARY PRINCIPLES

It is difficult to offer a precise definition of the precautionary principle as many different versions of it exist. The differences between these versions can be radical. The precautionary principle has been conceptualized as a *rule of choice* intended to help us select the best course of action, as an *epistemic rule* intended to guide only our beliefs and as a *procedural requirement* specifying processes that policymakers should follow when making risky decisions.16
Ahteensuu, M., & Sandin, P. (2012). The precautionary principle. In Roeser S., Hillerbrand, R., Sandin, P., Peterson, M. (Eds.), *Handbook of risk theory: Epistemology, decision theory, ethics, and social implications of risk* (pp. 961–978). Dordrecht, The Netherlands: Springer.
 We treat the precautionary principle as a rule of choice. This is consistent with how the precautionary principle tends to be applied in the bioethics literature.17
Clarke, S. (2005). Future technologies, dystopic futures and the precautionary principle. *Ethics and Information Technology, 7*(3), 121–126.
 This is also the version of the precautionary principle that is most relevant to the motivating question of this article; i.e. how should we weigh the risks and benefits of human germline interventions?

Even restricting our analysis to the precautionary principle as a rule of choice, the precautionary principle can take many different forms. A distinction is commonly drawn between weak and strong forms of the precautionary principle. We draw a parallel distinction between negative and positive precautionary principles.18
Sandin and colleagues label these the ‘argumentative' and ‘prescriptive' precautionary principles, respectively. Sandin P., Peterson, M. Hansson. S. O., Rudén, C., & Juthe, A. (2002). Five charges against the precautionary principle. *Journal of Risk Research, 5*(4), 287–299. What we call the negative precautionary principle has also been described as the ‘meta‐precautionary principle' – a meta‐rule intended to disqualify certain approaches to decision‐making. Steel, D. (2013). The precautionary principle and the dilemma objection. *Ethics, Policy & Environment, 16*(3), 321–340.
 This distinction runs as follows:

**Negative precautionary principle**. When an activity may cause harm we should not abstain from taking precautionary action because we lack certainty that the activity in question would cause harm.

**Positive precautionary principle**. We should take (some form of) precautionary action against activities that may cause (some kinds of) harm.

Negative versions of the precautionary principle do not make positive claims about when precautionary action should be taken; they merely reject one possible reason against taking precautionary action. By contrast, positive precautionary principles require decision‐makers to take certain kinds of precautionary measures against certain kinds of threats. Positive versions of the precautionary principle can range from being extremely demanding to being extremely undemanding, depending on what kinds of threats are thought to trigger the principle, what kinds of remedies are prescribed and how strongly these remedies are recommended.19
Sandin, P. (1999). Dimensions of the precautionary principle. *Human and Ecological Risk Assessment, 5*(5), 889–907.
 Consider the following two hypothetical positive precautionary principles:

**Strong positive precautionary principle**. One must take extensive precautionary action to eliminate fully *any* potential threats to human well‐being, regardless of the costs of the precautionary action, the likelihood that the threat would eventuate and the degree of harm posed by the threat.

**Weak positive precautionary principle**. One should consider taking some minimal precautionary actions against catastrophic threats to human well‐being that are highly likely to eventuate. 20
According to the standard distinction between ‘strong' and ‘weak' versions of the precautionary principle, these would both constitute ‘strong' versions of the precautionary principle (which might be misleading) or could perhaps be termed a ‘strong strong' and ‘weak strong' precautionary principle, respectively (which might be confusing).

Both the above precautionary principles are positive. However, where the first demands that extensive precautionary measures be taken under an extremely broad range of conditions, the second lightly recommends taking undemanding precautionary measures against only the most serious of hazards (as we presumably already do as a matter of course). The first positive precautionary principle is implausibly strong, whereas the second is unhelpfully weak. It is nonetheless possible that some moderate positive precautionary principle may provide a useful guide to decision‐making.

Some – although not all – forms of the precautionary principle also make explicit reference to the burden of proof.21
See, e.g. Manson, N. A. (2002). Formulating the precautionary principle. *Environmental Ethics, 24*, 263–274; Van den Belt, H., & Gremmen, B. (2002). Between precautionary principle and ‘sound science': Distributing the burdens of proof. *Journal of Agricultural and Environmental Ethics, 15*(1), 103–122; Cranor, C. F. (1999). Asymmetric information, the precautionary principle, and burdens of proof. In Reffensperger, C., & Tickner, J. *Protecting public health and the environment: Implementing the precautionary principle *(pp. 74–99), Washington, DC: Island Press; Harris, J., & Holm, S. (2002). Extending human lifespan and the precautionary paradox. *Journal of Medicine and Philosophy, 27*(3), 355–368.10.1076/jmep.27.3.355.298312187439
 Such versions of the precautionary principle generally require those advocating potentially hazardous activities to prove that the activity is (sufficiently) safe to be allowed; the corollary is that advocates of precautionary measures need not prove the activity is hazardous before implementing precautionary measures. We do not specifically address burden of proof requirements in this article. On one view, which we find convincing, burden of proof requirements are merely an instrumental means of achieving the precautionary principle's underlying normative goals – for example, to place greater weight on avoiding some kinds of outcomes than on others.22
Munthe, C. (2011). *The price of precaution and the ethics of risk*. New York, NY: Springer.



In what follows, we consider whether negative and positive versions of the precautionary principle should influence how we weigh the risks and benefits of GGE. We conclude that the precautionary principle does have a legitimate role to play in ethical analyses of GGE, although this role is less straightforward than many might expect. We reach two main conclusions: that no plausible version of the precautionary principle entails that we should reject outright the use GGE in human reproduction and that the precautionary principle can nonetheless help shape the developmental trajectory of GGE by encouraging some applications over others.

## 3 NEGATIVE PRECAUTIONARY PRINCIPLES

As described above, negative versions of the precautionary principle do not directly recommend any particular approach to decision‐making, but instead hold that certain kinds of arguments against precautionary measures should be rejected. Principle 15 of the Rio Declaration provides a canonical example. It asserts the following regarding possible threats to the environment:

[L]ack of full scientific certainty shall not be used as a reason for postponing cost‐effective measures to prevent environmental degradation. (Article 15 of the 1992 Rio Declaration on Environment and Development)

This version of the precautionary principle rejects a particular kind of argument against precautionary action: that because the existence of a particular threat has not been proven to a very high standard of evidence (i.e. full scientific certainty), we should not take precautionary measures against this threat. While the Rio Declaration's version of the precautionary principle focuses on threats of environmental degradation, it can just as easily be applied to threats to human health.

Although the Rio Declaration's precautionary principle has attracted criticism,23
See, e.g. Harris & Holm, op. cit., note 21.
 these criticisms seem to conflate the Declaration's minimal negative version of the precautionary principle with a much stronger positive version of the precautionary principle.24
Hughes, J. (2006). How not to criticize the precautionary principle. *Journal of Medicine and Philosophy, 31*(5), 447–464. See also Cambridge Quarterly of Healthcare Ethics. 2006;15(2):175‐83; discussion 184‐7. A paradox out of context: Harris and Holm on the precautionary principle. *Journal of Medicine and Philosophy, 31*, 184–187.10.1017/s096318010606021x16610756
 The Rio Declaration's precautionary principle is sound. Scientific certainty should not be a necessary condition for cost‐effective regulatory action against threats to the environment or to human health. It would be irrational not to take any action against potential threats whenever we lack full certainty that the anticipated harm will occur. Full certainty is virtually never achieved.

It is sometimes argued that negative versions of the precautionary principle are vacuous, in that they recommend rejecting arguments that it is already clear we ought to reject.25
See, e.g. Clarke, op. cit., note 17; Soule, E. (2000). Assessing the precautionary principle. *Public Affairs Quarterly, 14*(4), 309–328; Sunstein, C.R. (2005). *Laws of fear: Beyond the precautionary principle* Cambridge, UK: Cambridge University Press; Powell, R. (2010). What's the harm? An evolutionary theoretical critique of the precautionary principle. *Kennedy Institute of Ethics Journal, 20*(2), 181–206.10.1353/ken.0.031120653252
 While negative versions of the precautionary principle may be philosophically vacuous, they can nonetheless be pragmatically useful in policy debates. The history of regulatory (in)action against threats posed by lead, asbestos, cigarette smoke and greenhouse gas emissions – among other examples – shows that scientific uncertainty sometimes does forestall effective regulatory action against highly salient threats to the environment and public health, especially when this uncertainty is leveraged by interest groups opposed to tighter government regulation.26
See, e.g. Harremoës, P., Gee, D., MacGarvin, M., Stirling, A., Keys, J., Wynne, B., … Guedes Vaz, S. (2013). *The precautionary principle in the 20th century: Late lessons from early warnings* London, UK: Routledge; Oreskes, N., & Conway, E. M. (2010). *Merchants of doubt: How a handful of scientists obscured the truth on issues from tobacco smoke to global warming* 1st US edn. New York, NY: Bloomsbury Press; Percival, R. V. (2005). Who's afraid of the precautionary principle. *Pace Environmental Law Review, 23*(1), 21–81.
 Negative versions of the precautionary principle may therefore have a legitimate role to play in contexts where less‐than‐certain threats are liable to the overlooked.

Although sometimes pragmatically useful, the negative precautionary principle has little to contribute to the topic of GGE. The negative precautionary principle would serve to reject the following claim: that we should allow GGE unless threats to future generations are established to the level of full scientific certainty. This is not a particularly useful contribution to the debate, as few (if any) commentators have actually made this kind of claim. Instead, the current debate focuses largely on whether heritable germline modification should be rejected outright – as is currently the case in many jurisdictions27
Isasi, R., Kleiderman, E., & Knoppers, B. M. (2016). Editing policy to fit the genome? *Science, 351*(6271), 337–339.10.1126/science.aad677826797999
 – or conversely, whether we should allow some forms of germline modification *once the technology has met some appropriate threshold for safety*. Almost every major group, organizational and government statement favouring GGE has emphasized that safety and efficacy issues will need to be resolved before GGE is used in human reproduction.28
Ormond, K. E., Mortlock, D. P., Scholes, D. T., Bombard, Y., Brody, L. C., Faucett, W. A., …, Young, C..E. (2017). Human germline genome editing. *American Journal of Human Genetics, 101*(2)*,*167–176.10.1016/j.ajhg.2017.06.012PMC554438028777929
 There seems to be little danger of the potential risks of GGE being excluded from consideration.

It is worth reiterating that the scope of the negative precautionary principle is very narrow. It serves only to reject the requirement that we are *fully certain* that a threat exists before enacting precautionary measures. The negative precautionary principle does not entail that we require absolute certainty that GGE is safe before modifying the human germline. Indeed, the negative precautionary principle does not prescribe any specific method for weighing the possible costs and benefits of GGE. For this, we would need to turn to the positive precautionary principle.

## 4 POSITIVE PRECAUTIONARY PRINCIPLES

Unlike negative precautionary principles, positive precautionary principles prescribe a specific approach to decision‐making when we face threats to human health or the environment. Both the kind of threats that trigger such versions of the precautionary principle and the prescribed regulatory response can vary depending on how the principle is fleshed out; accordingly, positive precautionary principles can take a huge variety of forms. However, one common feature of positive precautionary principles is that they are generally understood in opposition to standard cost‐benefit analyses. A standard cost‐benefit analysis recommends that we take whichever course of action has the highest expected utility (which can be defined, roughly, as the sum of the various costs and benefits associated with that course of action, adjusted for the probability these costs and benefits will be realized). Positive precautionary principles recommend that we replace or supplement cost‐benefit analyses with a more explicitly precautionary approach to risk management. The key question, then, is whether such a departure from a cost‐benefit analysis can be justified.

Positive precautionary principles have been criticized on two grounds: for being unreasonably conservative and for being fundamentally incoherent. We explore both criticisms below. We then outline some positive precautionary principles that are not vulnerable to these objections. In what follows we refer to positive precautionary principles simply as ‘the precautionary principle' or ‘precautionary principles'; we continue to refer to negative precautionary principles as such.

### 4.1 THE CONSERVATIVISM OBJECTION

The conservativism objection holds that the precautionary principle undermines human well‐being by placing too high a barrier on technological progress. One version of the conservativism objection holds that human well‐being is better served by existing forms of cost‐benefit analysis;29
Burnett, H. S. (2009). Understanding the precautionary principle and its threat to human welfare. *Social Philosophy and Policy, 26*(2), 378–410; Miller, H. I., & Conko, G. (2001). Precaution without principle. *Nature Biotechnology, 19*(4), 302–303, Star, C. (2003). The precautionary principle versus risk analysis. *Risk Analysis, 23*(1), 1–3.
 another holds that we should replace the precautionary principle with a ‘proactionary principle' that privileges the protection of industry's freedom to innovate over the avoidance of risks posed by technological progress.30
More, M. (2005). The proactionary principle. In M. More & N. Vita‐More (Eds.), The transhumanist reader: Classical and contemporary essays on the science, technology, and philosophy of the human future (pp. 258–267). New York, NY: Wiley & Sons.
 Both versions of the conservativism objection reject the precautionary principle on the grounds that it is unreasonably conservative.

This criticism is not fundamental to the precautionary principle. The complaint here seems to be that proponents of precautionary principles place too much weight on avoiding one set of risks (for example, the risk of environmental degradation) relative to another set of risks (for example, the risk of lost gains caused by rejecting potentially beneficial technologies). Yet neither the precautionary principle in general nor the specific versions critiqued by these authors requires that we privilege the risks associated with novel technologies over risks associated with government regulation. Consider the 1988 Wingspread Statement, which has been criticized by many of the above authors for being unreasonably conservative:

When an activity raises threats of harm to the environment or human health, precautionary measures should be taken even if some cause and effect relationships are not fully established scientifically.[…] The process of applying the precautionary principle must […] involve an examination of the full range of alternatives, including no action.31
Ashford, N., Barrett,K., Bernstein, A., Costanza, R., Costner, P., Cranor, C., … Warledo. J. (1998). *Wingspread statement on the precautionary principle* Retrieved from http://www.who.int/ifcs/documents/forums/forum5/wingspread.doc

For a stronger positive precautionary principle, consider the ‘Final Declaration of the First European “Seas at Risk” Conference, 1994:

If the ‘worst case scenario' for a certain activity is serious enough then even a small amount of doubt as to the safety of that activity is sufficient to stop it taking place.32
Quoted in Sunstein, op. cit., note 25, p. 20.

In principle, these formulations of the precautionary principle could be applied to both the risks associated with the current status quo and the risks associated with government regulation. Indeed, the Wingspread Statement explicitly stipulates that policymakers examine the full range of policy alternatives (including no action); the threat of lost gains could easily form part of this deliberation. Moreover, if a prohibition or moratorium on some potentially beneficial technology threatens the environment or human health, these precautionary measures could themselves be criticized using the precautionary principle. The conservativism objection, then, does not amount to a complaint about the precautionary principle per se but is rather a complaint about the biased way that the principle's opponents believe it tends to be applied.

There is, however, a potential problem with this response to the conservativism objection. One might think that applying the principle to every possible course of action (including precautionary measures themselves) would either result in the precautionary principle sometimes blocking *every* option available to us (rendering the principle incoherent) or cause the precautionary principle to collapse into standard cost‐benefit analyses (rendering the principle superfluous). We consider both concerns in the following section.

### 4.2 THE INCOHERENCE AND SUPERFLUITY OBJECTIONS

The most serious criticism of the precautionary principles holds that it will give conflicting guidance if consistently applied, rendering the principle incoherent.33
Clarke, op. cit., note 17; Harris & Holm, op. cit., note 21; Sunstein, ibid.
 The incoherence objection is most commonly levelled against strong positive versions of the precautionary principles. Sunstein, for example, focuses his criticisms on a hypothetical version of the precautionary principle that requires us to abstain from courses of action that pose threats to health, safety, or the environment, regardless of the costs of precautionary action or the likelihood that the harm will eventuate. Sunstein charges that the precautionary principle (so understood) is incoherent, as it will often rule out *every *possible course of action. For example, it is likely that if we fail to reduce greenhouse gas emissions, climate change will contribute to a significant number of deaths by the end of the century. This is a serious harm and so should trigger the precautionary principle. At the same time, it is possible that reducing emissions will cause social and economic changes that reduce the well‐being of many people. This would *also* constitute serious harm and so should also trigger Sunstein's precautionary principle. The precautionary principle (so understood) thus entails – paradoxically – both that we should reduce greenhouse gas emissions and that we should abstain from reducing them. For Sunstein, then, the precautionary principle should be rejected not because it ‘leads in bad directions, but because read for all it is worth, it leads in no direction at all'.34
Sunstein, ibid., p.14.



The incoherence objection holds even if one narrows the scope of the precautionary principle to apply to only a small set of threats. Consider, for example, anti‐catastrophe versions of the precautionary principle, which only preclude courses of action that pose plausible threats of catastrophic harm.35
Manson, op. cit., note 21; Sunstein, ibid.
 Narrowing the precautionary principle in this way does not resolve the incoherence objection, for in some contexts taking precautionary measures against threats of catastrophic harm may *also *carry the threat of catastrophic harm. For example, while the current trajectory of climate change is likely to cause catastrophic harms, one could also speculate that sharply reducing emissions could *also *cause catastrophic harms ‐ for example, if the economic sacrifices required to reduce emissions contribute to political destabilization, thereby increasing the risk of devastating nuclear war. Assuming both scenarios are plausible, both would be disallowed under anti‐catastrophe versions of the precautionary principle.36
Manson, ibid.
 Even if the anti‐catastrophe principle would not have paradoxical implications in this specific context (because, for example, the threat of nuclear war is not sufficiently plausible to trigger the anti‐catastrophe principle), there are presumably at least *some *contexts where anti‐catastrophe principles would foreclose all possible courses of action, rendering the principle incoherent.

Can the paradoxical implications of the precautionary principle be addressed short of rejecting the principle itself? One strategy might be to specify that we should undertake only the precautionary measures that can be justified using standard cost‐benefit analyses.37
We are assuming here that standard cost‐benefit analysis would recommend the option with the highest expected utility, but this is not necessarily true of current scientific practices. One minimal version of the precautionary principle (which we do not discuss here) recommends adjusting our scientific practices towards risk neutrality when they inadvertently promote risk‐taking behaviour. See Hansson, S. O. (1999). Adjusting scientific practices to the precautionary principle. *Human and Ecological Risk Assessment, 5*(5), 909–921.
 Some proponents of the precautionary principle have taken this approach. For example:

Where there are significant risks of damage to the public health, we should be prepared to take action to diminish those risks, even when the scientific knowledge is not conclusive, if the balance of likely costs and benefits justifies it.38
Horton, R. (1998). The new new public health of risk and radical engagement. *Lancet, 352*(9124), 251–252.10.1016/S0140-6736(05)60254-19690400

Narrow versions of the precautionary principle such as the anti‐catastrophe principle could likewise be amended to specify that any precautionary measures taken against catastrophic harms should be consistent with a cost‐benefit analysis.39
Sunstein – who defends an anti‐catastrophe version of the precautionary principle – offers some qualifications to his anti‐catastrophe principle that can be interpreted in this way. For example, Sunstein stipulates that precautionary measures against potentially catastrophic risks should be proportional to both the probability and magnitude of the potential harm, thereby incorporating aspects of cost‐benefit analysis. Sunstein, op. cit., note 25, Chapter 5.
 Does this provide an adequate response to the incoherence objection?

It does not. Although such versions of the precautionary principle sidestep the incoherence objection, this comes at a steep cost. The suggestion that we apply a standard cost‐benefit analysis would seem to obviate the need for the precautionary principle in the first place – for if the precautionary principle ultimately just prescribes a standard cost‐benefit analysis, we might as well call for a cost‐benefit analysis directly. If the precautionary principle is to make a distinct contribution to bioethical debates – including the debate on GGE – it should justify some departure from standard cost‐benefit analyses. What is needed, then, is a plausible rationale for placing extra weight on specific kinds of risks when we decide what course of action we should take. The remainder of this article outlines and evaluates specific versions of the precautionary principle that might be able to justify such a departure from cost‐benefit analysis.

## 5 TAKING PRECAUTIONS TO CORRECT FOR BIAS

One justification for the precautionary principle holds that it may provide a valuable tool to counteract cognitive biases. The field of behavioural psychology provides compelling evidence that our cognitive processes are vulnerable to a range of predictable biases, leading to predictable errors in judgement.40
See, e.g. Kahneman, D. (2011). *Thinking, fast and slow*. New York, NY: Farrar, Straus and Giroux.
 This suggests a potential role for the precautionary principle: to correct for our cognitive bias by placing extra weight on the avoidance of threats that we are liable to undervalue. For example, Dana argues that two cognitive biases contribute to widespread reluctance to take serious action against climate change: an irrational tendency to weigh certain costs (such as higher energy bills) more heavily than uncertain costs (such as possible catastrophes associated with runaway climate change) and an irrational tendency greatly undervalue harms we would incur far in the future compared with harms we would incur in the present. On Dana's view, the precautionary principle could help counteract these tendencies. The trick would be to develop a version of the precautionary principle that artificially places enough extra weight on avoiding threats we are prone to undervalue, thereby cancelling out forms of human irrationality that would otherwise go unchecked.41
Dana, D. (2009). The contextual rationality of the precautionary principle. *Queen's Law Journal, 35*, 67.



If our attitudes towards GGE are skewed by irrational biases, then it might make sense to try to counterbalance such biases via some suitably designed version of the precautionary principle. The difficulty, however, is that many cognitive biases could plausibly influence our views on germline modification, and not all of them would necessarily weigh in the same direction. For example, the familiarity bias – i.e. a tendency to be more worried by unfamiliar risks than familiar ones – might cause us to be unduly worried by the novel threats posed by GGE. Similarly, according to status quo bias there is likely to be a bias against GGE, as the weight of current opinion is against its use. On the other hand, temporal myopia – i.e. the tendency to discount greatly the long‐term effects of our decisions relative to short‐term effects – might lead us to place undue weight on the short‐term benefits of GGE (for example, in opening up new reproductive options) relative to potential harms that might befall future generations. Pending further analysis, it is unclear whether the various cognitive biases to which we are susceptible would cumulatively weigh for or against germline modification and therefore it is also unclear what role (if any) exists for a bias‐corrective precautionary principle.

## 6 TAKING PRECAUTIONS AGAINST VIOLATIONS OF NEGATIVE DUTIES

Weckert and Moor defend a version of the precautionary principle that places greater weight on the goal of avoiding threats of harm relative to the goal of achieving possible benefits. They point out that negative duties not to inflict harm are generally considered more stringent than positive duties to do good. One standard example holds that it is usually considered worse to drown a child than to fail to save a child that happens to be drowning. By extension – and *pace *standard cost‐benefit analysis – if we are trying to decide whether to embark on a risky course of action, the risk that we will harm others through our actions should loom especially large. Accordingly, Weckert and Moor suggest that we should not simply choose the course of action with the highest expected utility, but instead place extra weight on avoiding violations of our negative duties towards others.42
Weckert, J., & Moor, J. (2006). The precautionary principle in nanotechnology. *International Journal of Applied Philosophy, 20*, 191–204. An alternative route to a similar destination might be to place more weight on avoiding harms than on achieving benefits, relative to some baseline for a *prima facie *acceptable balance of risks and possible benefits. See Munthe, op. cit., note 22.



Three caveats are in order. Firstly, Weckert and Moor's version of the precautionary principle relies on there actually being some moral distinction between positive and negative duties. However, this distinction is controversial.43
Russell, B. (1977). On the relative strictness of negative and positive duties. *American Philosophical Quarterly, 14*(2), 87–97; Persson, I., & Savulescu, J. (2012). *Unfit for the future: the need for moral enhancement*. Oxford: Oxford University Press.11664855
 Secondly, even if positive duties do generally outweigh negative duties, we would still need to determine how much extra weight we should place on avoiding threats of harm relative to pursuing possible benefits. Thirdly, applying Weckert and Moor's version of the precautionary principle to reproductive technologies (such as GGE) may raise the non‐identity problem, as the potential harms of employing such technologies are likely to fall on people who would not have existed if these technologies had not been employed.44
The use of GGE may change whether prospective parents conceive using natural methods or genetic selection, the timing of gamete retrieval, and/or the screening of gametes. Any such changes would likely result in a different embryo being created from that in the absence of GGE. Equally, the descendants of these persons would presumably not have been created in the absence of GGE.
 Briefly, the non‐identity problem points towards the difficulty of explaining why it is wrong to bring people into existence who will experience harm if (a) the harm is not so bad that it renders life not worth living, and (b) this harm could be avoided only by bringing a different person into existence instead. Although some argue that we have a negative duty against inflicting the kind of non‐person‐affecting harms described by the non‐identity problem,45
See, e.g. Brock, D. W. (1995). The non‐identity problem and genetic harms – the case of wrongful handicaps. *Bioethics, 9*(3–4), 269–275.10.1111/j.1467-8519.1995.tb00361.x11653042
 there is no consensus on this point.46
See, e.g. Boonin, D. (2008). How to solve the non‐identity problem. *Public Affairs Quarterly, 22*(2), 129–159.
 All three issues would need to be resolved before Weckert and Moor's version of the precautionary principle can be applied to GGE.

## 7 TAKING PRECAUTIONS AGAINST POORLY UNDERSTOOD RISKS

Ordinary cost‐benefit analysis is designed to deal with situations where the probabilities of various outcomes are well understood. However, we may not always know what threats are posed by a potentially hazardous activity or we may lack an adequate basis from which to estimate the likelihood that particular threats will eventuate. Such circumstances pose a serious challenge for cost‐benefit analysis, as such analysis requires that we can at least estimate the expected utility of the various courses of action open to us. One version of the precautionary principle known as the ‘Rawlsian core precautionary principle' (RCPP) is designed to deal with precisely those contexts where we cannot assign probabilities to the possible outcomes of our actions and where cost‐benefit analysis is therefore unfeasible or highly unreliable.

The RCCP, as defined by Gardiner, species a set of circumstances in which we should follow a Maximin decision‐making rule, according to which we should choose the course of action with the least bad worst‐case scenario. Although Gardiner does not recommend the maximin principle as a general‐purpose decision rule, he *does *recommends following the maximin principle when the following conditions are jointly met: (a) there is a plausible threat of significant harm, (b) decision‐makers lack reliable information about the probabilities of the possible outcomes of their actions, and (c) we are relatively indifferent towards the potential gains we would forgo by following the maximin strategy (at least compared with the risks that the Maximin strategy would avert).47
Gardiner, S. M. (2006). A core precautionary principle. *Journal of Political Philosophy, 14*(1), 33–60. For a related argument (that does not specifically mention the precautionary principle), see Hansson, S. O. (1996). Decision making under great uncertainty. *Philosophy of the Social Sciences, 26*(3), 369–386. Other formulations of the precautionary principle have applied the maximin rule without explicitly limiting its application to conditions of uncertainty. See, e.g. Hansson, S.O. (1997). The limits of precaution. *Foundations of Science, 2*(2), 293–306. As discussed below, we believe such formulations of the precautionary principle can give too much priority to avoiding worst‐case scenarios.



The RCPP sidesteps the incoherence objection by clearly recommending just one of the available policy alternatives: that which has the least bad worst‐case outcome. Moreover, in addition to being intuitively plausible, the idea that we should follow the Maximin principle under the sorts of circumstances outlined by the RCPP already plays a well‐established role in political philosophy.48
Rawls, J. (2005). *A theory of justice*. Cambridge, MA: Belknap Press.
 Notably, however, the RCPP can provide guidance only in scenarios where the probabilities of harm or benefit are highly uncertain; it is not equipped to deal with scenarios where probabilities are relatively clear. So while the RCPP may be applicable to GGE while the risks remain uncertain and unquantified, the RCPP will become increasing irrelevant as future research improves our ability to anticipate the risks and benefits of GGE. If we want to replace cost‐benefit analysis with something more explicitly precautionary – and not just adopt the precautionary principle in contexts where a cost‐benefit analysis cannot be applied – we will need to turn to some other variety of the precautionary principle.

Firstly, however, it is worth explaining why the Maximin principle (and therefore the RCPP) should not be followed when we can assign probabilities to the possible outcomes of our actions: because the Maximin principle is implausibly loss‐averse in such contexts. Imagine that states of affairs can vary from 0 units of health (death) to 100 units. X is at level 10. If intervention GE* is employed, there is a 1% chance of losing 1 unit of welfare but a 99% chance of gaining 90, that is, achieving perfect health. Such a risk is almost certainly worth taking, even though maximizing the minimum entails doing nothing. To follow the Maximin principle in this context would give too much priority to the worst‐case scenario.

## 8 A SUFFICIENTARIAN PRECAUTIONARY PRINCIPLE

In line with sufficientarianism in distributive justice,49
Crisp, R. (2003). Equality, priority, and compassion. *Ethics, 113*(4), 745–763; Frankfurt, H. (1987). Equality as a moral ideal. *Ethics, 98*(1), 21–43; Savulescu, J. (2002). How do we choose which life to save? Equality of access or a fair go? *Current Paediatrics, 12*(6), 487–492; Shields, L. (2012). The prospects for sufficientarianism. *Utilitas, 24*(4),101–117.
 we can conceive of a sufficientarian precautionary principle (SPP). According to sufficientarianism we should choose that option that has most people (or provides the greatest chance to be) above the level of a sufficiency threshold, or a ‘fair go'. According to the SPP we should take precautions against threats to achieving or maintaining a sufficient level of well‐being. The SPP will sometimes recommend the same kinds of measures recommended by other versions of the precautionary principle, which include bans, moratoria, premarket testing or requests for extra scientific information before proceeding on a potentially dangerous course of action.50
Ahteensu & Sandin, op. cit., note 16; Sandin, op. cit., note 19.



As in the previous example, imagine lives vary from 0–100 units and 80 represents the threshold for a decent life, one that is sufficiently good or that constitutes a fair go. According to sufficientarianism, we should distribute resources to bring as many people as possible who are below the sufficiency level (80) above it. According to the SPP we should avoid options which put (or risk putting) more people below the sufficiency level.

Imagine X is at 95 units. GE** has a 99% chance of raising X to 100, but a 1% chance of killing her. SPP requires we avoid GE**, for it is not worth even a small chance of death when one is doing so well. A standard cost‐benefit analysis would require employing GE** as the expected utility of doing nothing is 95 versus 99 of employing the intervention. But this is arguably too insensitive to low risks of large losses – we stand to lose too much to employ gene editing in this circumstance.51
We think the SPP can help show why He Jiankui's experiment was unethical. He Jiankui used gene editing to attempt to make two babies, Lulu and Nana, resistant to HIV. As the embryos used in this experiment were already healthy, the resulting children would presumably have enjoyed a sufficient level of well‐being if gene editing were not performed. According to the SPP, we have especially strong reasons against exposing children to the risks of gene editing (which might cause them to fall below a sufficient level of well‐being) if gene editing is unnecessary to secure a sufficient level of well‐being.



But now compare X with Y, who is at 50 units. There is a 99% chance of raising Y to 100 with GE***, but a 1% chance of killing her. In this case, the intervention may be worth trying. Indeed, according to SPP, we should employ GE*** in this circumstance.

The SPP, then, would place especial weight on avoiding threats that would place people below a sufficient level of well‐being. On this view, GGE might be worth undertaking when the level of well‐being without intervention is low. This is most likely to be the case when GGE is used to correct catastrophic genetic abnormalities.

Is the SPP properly understood as a version of the precautionary principle? Admittedly, it adopts a non‐traditional threshold for taking precautionary action. Where other versions of the precautionary principle commonly recommend taking precautionary action against threats of serious, catastrophic or irreversible harm,52
Ahteensu & Sandin, op. cit., note 16; Persson, E. (2016). What are the core ideas behind the precautionary principle? *Science of the Total Environment, 557*, 134–141; Sandin, op. cit., note 19.10.1016/j.scitotenv.2016.03.03426994801
 the SPP recommends taking precautionary action against threats to achieving or maintaining a *sufficient* level of well‐being. However, like other versions of the precautionary principle, the SPP calls us to depart from a standard cost‐benefit analysis when we face particular kinds of risks or threats. This kind of departure from standard cost‐benefit analyses is arguably *the* defining feature of positive versions of the precautionary principle,53
Randall, A. 2011. *Risk and precaution* Cambridge, UK: Cambridge University Press; Clarke, S. (2013). The precautionary principle and the dual‐use dilemma. In Rappert, B., & Selgelid, M. J. (Eds.), *On the dual uses of science and ethics: Principles, practices, and prospects *(pp. 221–231). Canberra, Australia: ANU Press; Clarke, op. cit., note 17; Dana, op. cit., note 41.
 which, in our view is sufficient to render the SPP a version of the precautionary principle.

## 9 TAKING PRECAUTIONS TO PROMOTE HEALTH SECURITY

Standard cost‐benefit analyses recommend taking the course of action with the highest expected utility (i.e. the weighted average of the utilities of each possible outcome). It is insensitive to the degree of variance among the possible outcomes of our actions; an option that could cause either great benefit or catastrophic harm may have the same expected utility as an option that could cause (at best) a slight benefit or (at worst) slight harm. This risk insensitivity is arguably problematic, as we may have moral reason to prefer policies that minimize grave risks to public health independently of these risks' contribution to expected utility.

There are at least two possible reasons why it might be reasonable to be risk averse with respect to public health. Firstly, health security may be intrinsically valuable. Consider the following scenario, which arguably provides some intuitive grounds to think that health security matters for its own sake:

If two (possible) societies/worlds are exactly similar […] except that one of the societies/worlds is highly vulnerable in a way that the other is not (imagine that, for example, unbeknownst to the inhabitants, the former is surrounded by meteors that might, as a result of random chance, impact and destroy it at any moment, while the latter is not threatened by such meteors), then it might be reasonable to think the latter is a(n intrinsically) better society/world (even if the disaster in question never eventuates).54
Selgelid, M. J. (2013). Biodefense and dual‐use research: The optimisation problem and the value of security. *Journal of Medical Ethics, 39*(4), 205–206, p. 205.10.1136/medethics-2012-10092323139390

Following Selgelid, if we believe ex post that the second world was better off than the first (even though the disaster never eventuated), then we appear to value security for its own sake. And if we are right to hold that security is intrinsically valuable, then we ought to be at least somewhat risk averse when making decisions that affect public health. This is because gambling with public health would undermine something of intrinsic value (i.e. health security), even if this gamble is neutral with respect to expected utility.

Secondly, health security may be instrumentally valuable. This is because grave risks to population health can undermine policymakers' ability to plan for the future. The more insecure the future health and life expectancy of the population becomes, the more difficult it will be to anticipate the population's future health needs or predict the effects of social and economic policies. To take a simple example, it would be difficult to develop policies that anticipate a population's future health needs if there is a significant chance that life expectancy could either increase or drop precipitously in the coming years. Because threats to public health undermine social planning, we have instrumental reasons to prefer less risky policies to highly risky ones.55
Herington J. (2016). Health security and risk aversion. *Bioethics, 30*(7), 479–489.10.1111/bioe.1225526990349



If health security is intrinsically and/or instrumentally valuable, then we should not make decisions that could affect public health *purely *by choosing the option with the highest expected utility. Instead, we ought to place some independent weight on avoiding significant threats to health security (above and beyond these threats' negative contribution to expected utility). This weighting of security is captured by some existing versions of the precautionary principle aimed at reducing risks of losses that could be much larger than the potential gains.56
See, e.g. Randall, op. cit., note 53.
 This weighting of security is also largely captured by the SPP described above, which places extra weight on avoiding threats that would cause individuals to fall below some sufficient level of well‐being.57
We leave open the question of whether the SPP fully captures the appeal of other risk‐aversive precautionary principles.



In the context of GGE, a risk‐aversive precautionary principle entails that we should consider not only whether GGE would promote expected utility, but also the impact of GGE on health security. In other words, we should be sensitive not only to the expected value of pursuing (or not pursuing) GGE, but also to the magnitude of the potential harms we would risk by taking this course of action. On the face of it, a concern for health security might seem to weigh against GGE, given the potential harms GGE may pose to future generations. In the following section of this article we show why this might not be the case.

## 10 A GENERAL CHALLENGE FOR PRECAUTIONARY APPROACHES TO GGE

In our view, the above versions of the precautionary principle are not vulnerable to the standard objections to positive precautionary principles. They are not unduly conservative, they differ meaningfully from standard cost‐benefit analyses and, if properly applied, they do not lead to paradoxical conclusions. This is not to say that these forms of the precautionary principle are above question. However, given their prima facie plausibility, at a minimum they warrant further analysis

In this section, we want to draw attention to a general difficulty associated with applying the precautionary principle in the context of GGE. It might seem that insofar as GGE carries plausible and significant risks to future generations, most plausible versions of the precautionary principle would weigh against pursuing GGE. This is not necessarily the case, as *failing *to pursue GGE may also carry plausible and significant risks to future generations.

As described at the beginning of the article, one possible application of GGE is to eradicate recessive mutations and disease‐predisposing alleles. This would significantly improve the health of future generations; correspondingly, failing to use GGE for these purposes might indirectly threaten the health of future generations. In addition, consider a more direct argument for GGE, based on an argument recently advanced by Russell Powell: that unless we engage in GGE the population's genetic health will gradually decline, leaving future generations increasingly reliant on conventional medical technology. This is because (Powell argues) advances in conventional medicine have largely freed humankind from the pressures of natural selection, thereby leaving the human gene pool vulnerable to the accumulation of deleterious mutations. The risks of failing to engage in GGE are twofold. Firstly, future generations may need to dedicate substantial resources to correct for their poor state of genetic health, thereby tying up resources that could have been used to promote human well‐being in other ways. Secondly, as future generations become increasingly dependent on medical technology to achieve current levels of well‐being, they will also become increasingly vulnerable to catastrophic harm should conventional medicines become less readily available – for example, during a hypothetical future period of economic and political collapse. On Powell's view, GGE presents a valuable means of maintaining current levels of genetic health and shielding future generations from potentially catastrophic threats to their well‐being.58
Powell, R. (2015). In genes we trust: Germline engineering, eugenics, and the future of the human genome. *Journal of Medicine and Philosophy,* *40*(6), 669–695.10.1093/jmp/jhv02526475170



If Powell's analysis is correct, pursuing *and* failing to pursue GGE carry the sort of risks that might trigger (most of) the above precautionary principles. Both courses of action carry an extremely bad worst‐case scenario; both threaten to render public health less secure; both might cause people to fall below a sufficient level of well‐being; and both plausibly violate negative obligations to future generations.59
Abstaining from GGE might be said to violate a negative obligation not to (excessively) deplete natural resources on which future generations will rely, at least insofar as ‘natural resources' are understood to include genetic health.
 Viewed this way, the precautionary principles discussed in this article do not provide any clear guidance on whether GGE should be carried out.

In saying this, we are not making the well‐worn point that the precautionary principle can often be deployed both for and against the same policy. Although some versions of the precautionary principle can be used this way, these versions of the principle are vulnerable to the incoherence objection described earlier in this article and should therefore be rejected. Our argument is that the risks of pursuing and failing to pursue GGE seem largely symmetrical, which makes it practically difficult to determine what course of action the precautionary principle would ultimately recommend. It is therefore an open question whether the precautionary principle would weigh against GGE or whether it might (contrary to most commentators' expectations) actually *support* GGE.60
In some respects, our argument here parallels an argument advanced by some critics of the environmental movement: that the precautionary principle recommends that we loosen or eliminate policies designed to protect the environment, such as restrictions on greenhouse gas emissions. See, e.g. Goklany, I. M. (2001). *The precautionary principle: A critical appraisal of environmental risk assessment*. Washington, DC: Cato Institute. Although we do not necessarily endorse these arguments against environmental regulation, we agree that the precautionary principle can yield unexpected conclusions.



This is partly due to our limited understanding of the risks of GGE. As gene editing technologies are developed further it will become more possible to quantify the relevant risks, which may determine the appropriate way to deploy the precautionary principle. For example, the risks associated with off‐target mutations will become better understood as gene editing becomes more widely used in different cell types. The risks of on‐target mutations will become better understood as we understand more about genetics and genotype‐phenotype relation. The risks of accumulating germline mutations due to modern medicine could be better quantified using intergenerational studies to look at the rate at which mutations are actually accumulating and where in the genome they are accumulating. Therefore, although it is currently difficult to apply most versions of the precautionary principle to GGE, this is partly due to epistemic limitations that may be overcome in coming years.61
The Rawlsian core precautionary principle – discussed earlier in the article – is one exception. The RCPP is intended to apply to contexts where probabilities cannot be meaningfully assigned. It is therefore highly relevant to decision‐making while the risks of GGE are poorly understood.



Furthermore, the precautionary principle does offer relatively clear guidance on one point: prima facie, precautionary principles provide reasons to prefer some applications of GGE over others. Specifically, the precautionary principles described in this article provide a reason to favour GGE research focused on maintaining genetic health, enhancing the human species' long‐term viability62
Gyngell, C. (2012). Enhancing the species: Genetic engineering technologies and human persistence. *Philosophy & Technology*, *25*(4), 495–512.
 or negating existential risks63
See generally Persson & Savulescu, op. cit., note 43.
 over research aimed at modifying cosmetic traits and perhaps also over research aimed at promoting health beyond some sufficiently high level of well‐being. The latter practices – but not the former – would expose future generations to unbalanced long‐term risks of harm in exchange for short‐term benefits.

## 11 CONCLUSION

The precautionary principle aims to influence decision‐making in contexts where some human activity poses uncertain but potentially grave threats. This perfectly describes the controversy surrounding GGE. It is therefore surprising that the precautionary principle has received relatively little attention in the bioethics literature on gene editing. Where the precautionary principle has been discussed, it is generally assumed with minimal analysis that this principle would weigh against human germline modification and perhaps even rule it out altogether.64
See, e.g. Annas et al., op. cit., note 12; Peters, op. cit., note 15; Goldim, op. cit., note 15; Smith et al., op. cit., note 15.



We hope to have provided a more detailed sketch of the significance of different kinds of precautionary principle for GGE. We have argued that, while negative precautionary principles can be pragmatically useful in some contexts, they have little to contribute to the policy debate surrounding reproductive GGE. Positive precautionary principles are more closely relevant. Positive precautionary principles recommend placing especial weight on avoiding certain kinds of threats, such as threats we are cognitively primed to undervalue, threats that are poorly understood, threats to the achievement of a sufficient level of well‐being and threats to health security. While it is difficult to derive any straightforward policy recommendations from these positive versions of the precautionary principle, plausible versions of it would endorse GGE in at least some contexts – in particular, contexts where GGE could be used to correct otherwise catastrophic genetic mutations and/or to promote the long‐term robustness of human populations. Given that the precautionary principle is generally deployed *against *GGE, we think this is an important insight.

Much work remains to be done before the precautionary principle can yield concrete recommendations regarding GGE. We nonetheless hope to have made some headway in this article by showing that the precautionary principle should not be rejected outright, clarifying what role it might be able to play and drawing attention to some of the key questions that still need to be resolved.

